# The roles of employment status and income in the mental health of informal caregivers in Germany

**DOI:** 10.1186/s12889-024-20252-y

**Published:** 2024-10-14

**Authors:** Julia-Sophia Scheuermann, Anna Pendergrass, Katharina Diehl, Raphael M. Herr

**Affiliations:** 1 Centre for Health Services Research in Medicine, Department of Psychiatry and Psychotherapy, Uniklinikum Erlangen, Friedrich-Alexander-Universität Erlangen-Nürnberg (FAU), Schwabachanlage 6, 91054 Erlangen, Germany; 2 Professorship of Epidemiology and Public Health, Department of Medical Informatics, Biometry and Epidemiology, Friedrich-Alexander-Universität Erlangen-Nürnberg (FAU), 91054 Erlangen, Germany

**Keywords:** Employment, Informal caregiving, Mental health, Net household income

## Abstract

**Background:**

Informal caregivers often experience multiple negative consequences as a result of the informal care they provide. Among other factors, employment status, financial resources, and mental health are related to informal caregiving. This analysis examined the association between informal caregivers’ employment status and their mental health, as well as the moderating effect of net household income on this relationship.

**Methods:**

The research question was addressed with data from the German Socio-Economic Panel (SOEP) survey, comprising 3,053 informal caregivers (1,007 male; 2,046 female). Data were obtained through self-reports, and mental health was measured with the Summary Scale Mental Score. Stepwise adjusted multiple linear regression models were used to examine the association between employment status and mental health. The moderating effects were tested with interaction terms. All analyses were also stratified for gender.

**Results:**

Informal caregivers with full-time jobs reported better mental health than unemployed or marginally employed caregivers (β = 0.077, *p* < 0.001). The significant interaction term for full-time (β=-0.066, *p* = 0.001) and part-time workers (β=-0.066, *p* = 0.003) indicated a moderating effect of net household income on the association between employment status and mental health. This finding was especially evident in women.

**Conclusions:**

Employment appears to be a relevant protective factor for informal caregivers’ mental health. However, if informal caregivers are not employed, a low net household income might additionally restrict their mental health. Therefore, welfare policy structures must be created to reduce the negative financial consequences for informal caregivers and enable them to achieve work-life-care balance.

## Introduction

The German labor market is facing extensive challenges due to demographic changes and the associated increase in the need for informal care. Informal care comprises unpaid care provided to individuals who need assistance due to illness, disability, age, or other conditions, which is typically provided by family, friends or other persons within the context of an existing relationship [1]. According to employment prognoses by the Federal Statistical Office in Germany based on the current retirement age (67 years), around a quarter of the population will no longer be employed by 2040 [2]. In addition, the industry is experiencing a shrinking of the labor force and a shortage of skilled workers due to demographic changes due to continuously declining fertility rates and simultaneously high life expectancy [3] as well as job changes that are due to digitalization [4]. Another limiting factor for the number of skilled workers is the lack of compatibility between work and informal care duties [5]. On the one hand, a care-related reduction or cessation of employment can decrease the number of skilled workers [6]. In particular, as the intensity and burden of care increases, informal caregivers experience a time conflict with their employment and other social roles [7]. As a result, informal caregivers often reduce their employment or even immediately abandon it. On the other hand, the health consequences of informal caregiving might also contribute to a decline in the caregiver’s ability to work and a reduction in working hours or even terminate their employment completely.

Providing informal care is often associated with negative impacts on the physical and mental health of informal caregivers [7, 8]. Mental health consists of functional and dysfunctional components and comprises according to the WHO mental well-being enabling people to cope with their lives [9]. An incompatibility of various social roles causes increased stress (emotional, social, and financial) and strain [7, 10], which can also be associated with negative impacts on the health of informal caregivers [10–13]. In addition to the amount of caregiving and work [14], the domestic living situation with the care recipient [15] has also been found to have a negative impact on mental health and emotional stress of informal caregivers.

Combining work and caregiving responsibilities seems burdensome. Studies on the influence of informal care and employment on the mental health of informal caregivers have shown negative associations [8, 10, 11]. The amount of caregiving was negatively associated with mental health, with employment being an additional stress factor for informal caregivers [8]. This negative association affects female caregivers in particular - both in terms of their mental health and their life satisfaction [16]. But for some, employment can serve as a buffer for the negative impacts of employment [7]. Thus, being unable to reconcile work and caregiving means emotional stress for informal caregivers [6], which can lead to burnout. Thereby, often gender differences result due to the fact, that women provide, in line with social stereotypes, the majority of informal care [17–21]. This means that women perform unpaid care work more frequently than men - a sign of the gender care gap that predominates in Germany [22, 23]. This is also one reason why women who live with a partner are less active in the labor market than men [24]. In consequence there is especially a negative impact on women’s mental health, e.g. more anxiety or a lower quality of life [12, 13, 25, 26]. However, after controlling for caregiving tasks and amount of time, there were no gender differences in depression or stress [26]. Thus, these negative effects do not necessarily have to be related to gender [27].

In their Informal Caregiving Integrative Model (ICIM), Gérain and Zech [28] argued that working while providing informal care might be a protective factor, as it relieves the caregiving strain by providing an emotional distraction. This positive aspect of relief through employment was also identified in the review by Neubert, König [7]. However, this effect should depend on several factors, including caregiving responsibilities, gender, and financial matters. Caregiving responsibilities are associated with a high level of stress and unpredictability as well as negative changes in family and relationships, which is consequently related with reduced mental health for informal caregivers [14]. This relationship is primarily influenced by the intensity of the informal caregiving activities [29], with higher effects for women [14].

Informal care can also be associated with financial consequences [29], which in turn may have a negative impact on mental health. In terms of work characteristics, a person’s financial situation can represent a stress factor. Nam [30] already provided evidence that financial difficulties are associated with a higher level of depressive symptoms. Experiencing financial stress increases caregivers’ vulnerability to subjective stress and burnout, which in turn increases their susceptibility to mental health problems [28, 31, 32]. In consequence, due to the influence of informal care on wages and the influence of wages on the mental health of these individuals, monthly net household income may be an effect modifier for the relationship between employment status and mental health of informal caregivers, which has not yet been investigated in the literature.

**Fig. 1:**
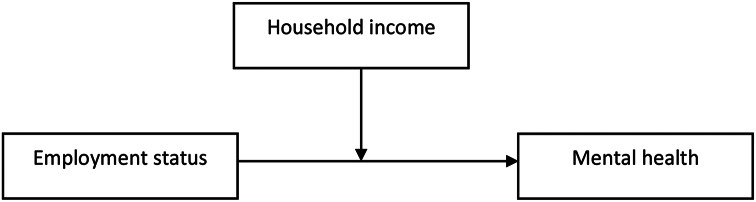
Schematic representation

Thus, the aim of this study was to examine the association between informal caregivers’ employment status and their mental health, while considering several determining factors. In particular, we tested for the moderating effect of monthly net household income on the association between employment status and mental health (Fig. 1). To consider potential gender differences, all analyses were stratified by gender.

## Methods

### Study design

This study used data from the German Socio-Economic Panel (SOEP) [33]. The SOEP constitutes a nationally representative household-based panel study in Germany with annual waves of measurement. The SOEP was made available by the German Institute for Economic Research (DIW) [34]. For this study, data from informal caregivers with essential information (i.e., mental health, employment status) were pooled due to the small sample size of each wave (waves: 2002, 2004, 2006, 2008, 2010, 2012, 2014, 2016, 2018), yielding an analytic sample of 3,053 informal caregivers (1,007 male; 2,046 female).

### Instruments

#### Mental health

Mental health was assessed with the predefined MCS Summary Scale Mental Score of the Short-Form 12v2 questionnaire (SF12). The MCS score is a standardized score based on the 2004 SOEP population. It has a mean of 50 and a standard deviation of 10 points with higher values indicating better mental health [35].

#### Employment status

The employment status of the informal caregivers was categorized into full-time, part-time, and not or marginally employed (including: training/apprenticeship, low-income earner, partial retirement with zero working hours, voluntary service, workshop for disabled people, and not gainfully employed).

#### Covariates

The level of long-term care comprised the following categories: errands outside the household, running the household, simple care tasks, and difficult care tasks. Gender was assessed as male or female, age is presented as age in years at time of the survey, and monthly net household income was disclosed in Euro (EUR).

### Statistical analysis

In a first step, descriptive analyses were computed for the total sample as well as for the gender subsamples. The associations between the informal caregivers’ employment status and their mental health were estimated in a second step with a series of linear regression models. The first model included only employment status as the independent variable. The second model additionally considered the level of care tasks, whereas the third model also included the sociodemographic factors (age, gender, and monthly net household income) and the survey year. The fourth and final model also included interaction terms between employment status and monthly net household income to test for potential moderating effects. All models were calculated for the total sample as well as separately for the male and female subsamples. The analyses were carried out with IBM SPSS Statistics (Version: 29.0.0.0), and the level of significance was defined as *p* < 0.05.

## Results

Most of the analytic sample was female (67%), and most of the informal caregivers were unemployed or marginally employed (73%, Table 1). In the male subsample, the proportion of unemployment or marginal employment was slightly higher (78%) than in the female sample (71%). The male sample was somewhat older (mean age = 65 years) than the female sample (mean age = 57 years). Most of the caregiving involved simple tasks (42%), followed by difficult tasks (31%). The mean monthly net household income was 2,646 Euro for the total analytic sample, 2,427 Euro for the male sample, and 2,754 Euro for the female sample.

**Table 1:** Sample description

		Total		Male		Female	
		Mean / %	*N*	Mean / %	*N*	Mean / %	*N*
Mental health scale		47.38	3,053	49.25	1,007	46.47	2,046
Employment							
Full-time		13.6%	415	19.6%	197	10.7%	218
Part-time		13.0%	396	2.5%	25	18.1%	371
Not/marginally		73.4%	2,242	78.0%	785	71.2%	1,457
Training/apprenticeship		0.3%	9	0.5%	5	0.2%	4
Low-income earner		5.5%	169	2.7%	27	6.9%	142
Partial retirement with zero working hours		0.4%	13	0.5%	5	0.4%	8
Voluntary service		0.1%	3	0.0%	0	0.1%	3
Workshop for disabled people		0.1%	2	0.2%	2	0.0%	0
Not gainfully employed		67.0%	2,046	74.1%	746	63.5%	1,300
Level of long-term care							
Errands outside the HH		8.4%	255	10.7%	108	7.2%	147
Running the HH		18.8%	574	22.0%	222	17.2%	352
Simple care tasks		42.0%	1,281	38.5%	388	43.6%	893
Difficult care tasks		30.9%	943	28.7%	289	32.0%	654
Age (years)		59.76	3,053	65.47	1007	56.95	2,046
Monthly net household income (EUR)		2,646.11	3,053	2,427.16	1007	2,753.87	2,046

*Note* HH = household, EUR = Euro

Table 2 presents the results of the regression analysis for the total sample. Compared with unemployed or marginally employed caregivers, those with part-time employment reported in the context of a statistical trend slightly better mental health (β = 0.032, *p* = 0.084), and those with full-time employment reported significantly better mental health (β = 0.077, *p* < 0.001; Table 2, Model 1). This association was independent of caregiving tasks (Model 2), sociodemographic factors (age, gender, net household income), or survey year (Model 3). The significant interaction terms (Model 4, Table 2) indicated a moderating effect of net household income on the association between employment status and mental health. Thereby, the association between employment status and mental health is weaker as the income increases.

**Table 2:** Results of the regression analyses, total sample (*n* = 3,053), outcome mental health scale (MCS)

Model						95% Confidence interval	
		*B*	Std. error	β	*p*	Lower bound	Upper bound
1	Constant	46.92	0.23		< 0.001	46.47	47.37
	Employment						
	Not/marginally	Ref.					
	Full-time	2.46	0.58	0.077	< 0.001	1.32	3,60
	Part-time	1.03	0.59	0.032	0.084	-0.14	2,19
2	Constant	47.24	0.33		< 0.001	46.60	47.88
	Employment						
	Not/marginally	Ref.					
	Full-time	2.24	0.58	0.070	< 0.001	1.09	3,38
	Part-time	1.01	0.59	0.031	0.088	-0.15	2,17
	Care tasks						
	Simple care tasks	Ref.					
	Errands outside the HH	0.65	0.75	0.016	0.383	-0.81	2,11
	Running the HH	0.70	0.55	0.025	0.198	-0.37	1,77
	Difficult care tasks	-1.54	0.47	-0.065	0.001	-2.45	-0,62
3	Constant	-281.10	74.06		< 0.001	-426.31	-135.88
	Employment						
	Not/marginally	Ref.					
	Full-time	1.55	0.63	0.048	0.014	0.31	2,78
	Part-time	1.08	0.64	0.033	0.092	-0.18	2,33
	Care tasks						
	Simple care tasks	Ref.					
	Errands outside the HH	0.49	0.73	0.012	0.505	-0.95	1,93
	Running the HH	0.22	0.54	0.008	0.688	-0.84	1,27
	Difficult care tasks	-1.64	0.46	-0.070	< 0.001	-2.55	-0,74
	Age (years)	0.03	0.01	0.048	0.020	0.01	0.06
	Survey year	0.20	0.04	0.096	< 0.001	0.12	0.27
	Female	-2.84	0.44	-0.122	< 0.001	-3.71	-1.98
	Monthly net household income	0.00	0.00	0.128	< 0.001	0.00	0.00
4	Constant	-276.39	73.90		< 0.001	-421.29	-131.50
	Employment						
	Ref: Not/marginally	Ref.					
	Full-time	2.02	0.66	0.063	0.002	0.73	3,31
	Part-time	1.68	0.69	0.052	0.015	0.33	3,03
	Care tasks						
	Simple care tasks	Ref.					
	Errands outside the HH	0.48	0.73	0.012	0.515	-0.96	1,91
	Running the HH	0.16	0.54	0.006	0.764	-0.89	1,21
	Difficult care tasks	-1.61	0.46	-0.068	< 0.001	-2.52	-0,71
	Age (years)	0.04	0.01	0.051	0.013	0.01	0.06
	Survey year	0.20	0.04	0.095	< 0.001	0.12	0.27
	Female	-2.85	0.44	-0.123	< 0.001	-3.71	-1.99
	Monthly net household income	0.00	0.00	0.178	< 0.001	0.00	0.00
	Interaction monthly net household income with:						
	Full-time	0.00	0.00	-0.066	0.001	0.00	0.00
	Part-time	0.00	0.00	-0.066	0.003	0.00	0.00

*Note* HH = Household; Monthly net household income in Euro (centered)

Table 3 presents the results of the gender stratified analyses. The general associations were evident in men and women; however, for men, there was no indication that net household income had a moderating effect.

**Table 3:** Results of the regression analyses, gender stratification, outcome: mental health scale (MCS)

			Male (*n* = 1,007)							Female (*n* = 2,046)					
Model							95% Confidence interval							95% Confidence interval	
			B	Std. error	β	*p*	Lower bound	Upper bound		B	Std. error	β	*p*	Lower bound	Upper bound
1	Constant		48.70	0.39		< 0.001	47.93	49.47		45.96	0.28		< 0.001	45.41	46.51
	Employment														
	Not/marginally		Ref.							Ref.					
	Full-time		2.34	0.88	0.084	0.008	0.62	4.07		1.92	0.78	0.055	0.014	0.39	3,44
	Part-time		3.62	2.24	0.051	0.106	-0.77	8.01		1.69	0.62	0.061	0.007	0.47	2,91
2	Constant		49.65	0.58		< 0.001	48.51	50.80		46.13	0.39		< 0.001	45.36	46.89
	Employment														
	Not/marginally		Ref.							Ref.					
	Full-time		2.21	0.87	0.079	0.012	0.50	3.93		1.69	0.78	0.049	0.031	0.16	3,22
	Part-time		3.86	2.23	0.054	0.083	-0.51	8.24		1.63	0.62	0.058	0.009	0.41	2,85
	Care tasks														
	Simple care tasks		Ref.							Ref.					
	Errands outside the HH		0.03	1.19	0.001	0.983	-2.32	2.37		0.42	0.95	0.010	0.663	-1.46	2,29
	Running the HH		-0.60	0.92	-0.023	0.514	-2.42	1.21		1.00	0.67	0.035	0.140	-0.33	2,32
	Difficult care tasks		-2.81	0.85	-0.115	0.001	-4.48	-1.14		-1.05	0.55	-0.045	0.058	-2.13	0,04
3	Constant		-163.59	132.71		0.218	-424.01	96.83		-346.43	89.55		< 0.001	-522.06	-170.80
	Employment														
	Not/marginally		Ref.							Ref.					
	Full-time		0.85	1.09	0.030	0.439	-1.30	3.00		1.34	0.81	0.038	0.098	-0.25	2,92
	Part-time		2.84	2.24	0.040	0.205	-1.55	7.24		1.10	0.67	0.039	0.101	-0.21	2,41
	Care tasks														
	Simple care tasks		Ref.							Ref.					
	Errands outside the HH		-0.07	1.19	-0.002	0.954	-2.39	2.26		0.60	0.94	0.014	0.522	-1.24	2,45
	Running the HH		-0.98	0.92	-0.037	0.285	-2.78	0.82		0.74	0.67	0.026	0.267	-0.57	2,05
	Difficult care tasks		-3.01	0.85	-0.123	0.000	-4.67	-1.35		-1.07	0.55	-0.046	0.051	-2.15	0,01
	Age (years)		-0.01	0.03	-0.008	0.825	-0.06	0.05		0.05	0.02	0.067	0.005	0.01	0.08
	Survey Year		0.10	0.07	0.048	0.160	-0.04	0.24		0.24	0.05	0.119	< 0.001	0.15	0.34
	Monthly net household income		0.00	0.00	0.149	< 0.001	0.00	0.00		0.00	0.00	0.126	< 0.001	0.00	0.00
4	Constant		-171.01	132.93		0.199	-431.86	89.84		-342.47	89.33		0.000	-517.64	-167.29
	Employment														
	Not/marginally		Ref.							Ref.					
	Full-time		1.14	1.10	0.041	0.302	-1.03	3.31		2.01	0.86	0.058	0.019	0.33	3,69
	Part-time		2.56	2.24	0.036	0.255	-1.85	6.96		1.78	0.72	0.064	0.014	0.36	3,19
	Care tasks														
	Simple care tasks		Ref.							Ref.					
	Errands outside the HH		-0.06	1.18	-0.002	0.959	-2.38	2.26		0.55	0.94	0.013	0.560	-1.29	2,39
	Running the HH		-1.04	0.92	-0.039	0.259	-2.84	0.77		0.65	0.67	0.023	0.332	-0.66	1,95
	Difficult care tasks		-3.04	0.85	-0.124	< 0.001	-4.70	-1.37		-1.03	0.55	-0.045	0.060	-2.10	0,04
	Age (years)		-0.01	0.028	-0.013	0.722	-0.066	0.046		0.05	0.02	0.072	0.002	0.02	0.08
	Survey year		0.10	0.07	0.048	0.160	-0.04	0.24		0.24	0.05	0.120	< 0.001	0.15	0.34
	Monthly net household income		0.00	0.00	0.180	< 0.001	0.00	0.00		0.00	0.00	0.183	< 0.001	0.00	0.00
	Interaction monthly net household income with:														
	Full-time		0.00	0.00	-0.070	0.070	0.00	0.00		0.00	0.00	-0.071	0.005	0.00	0.00
	Part-time		0.00	0.00	0.023	0.461	0.00	0.01		0.00	0.00	-0.080	0.004	0.00	0.00

*Note* HH = Household

## Discussion

The present analysis examined the relationship between employment status and mental health and the potential moderating effect of net household income in informal caregivers. Informal caregivers who also worked full-time jobs reported better mental health than those who were unemployed. The relationship between informal caregivers’ employment status and mental health was moderated by net household income.

In line with Fleitas Alfonzo, Taouk [29], the results confirm the relevance of employment status for the mental health of informal caregivers. In particular, caregivers who also had full-time jobs showed better mental health than those who were unemployed or were low-income earners, regardless of gender. For those working part-time, the result could be identified as a statistical trend. Consequently, informal care and employment are not only negatively linked but can also interact positively [7, 28, 36]. Indeed, employment can also serve as a buffer against the burden of informal caregiving and reduce role overload. Thus, employment can serve as a resource for the mental health of informal caregivers in terms of distraction and social interaction [37–39]. This finding is also in line with findings in the general population, for whom a protective effect of employment has also been demonstrated [40]. Because, according to the hypothesis of social causation, socio-economic status is directly related to mental health [41].

The net household income of informal caregivers is closely linked to their employment status. In the present study, in both the overall sample and the subsample of women, net household income significantly moderated the relationship between informal caregivers’ employment status and their mental health. This moderating effect did not emerge in the male subsample. Previous studies had not yet investigated this moderating effect directly. Liu, Dokos [36] did not find a moderating influence of financial burden on the relationship between employment status and subjective stressors. Such subjective stressors can in turn be seen as an indicator of possible mental health effects. However, analogous to our findings, Fleitas Alfonzo, Taouk [29] showed that the employment status of informal caregivers is linked to their mental health. Indeed, providing informal care can be associated with a person’s employment and income opportunities. Consequently, these opportunities relate to the financial well-being and mental health of informal caregivers. Therefore, net household income, which is closely linked to employment status, can be affected by the requirements of informal caregiving and consequently is positively related to the relationship between employment status and mental health of informal caregivers. Thus, informal caregivers may experience financial burdens and difficulties, which are also evident in the present sample, as the informal caregivers in this study earned two thirds of the mean net household income of people in Germany in 2021. Further, financial burdens are a common cause of stress and anxiety, which consequently affect the mental health of informal caregivers [42, 43]. Thus, net household income has an effect on the association of employment status and income. Nam [30] found that people with financial difficulties exhibited higher levels of depressive symptoms: Informal caregivers with financial difficulties reported more depressive symptoms than informal caregivers without financial difficulties. Thus, this finding indicates that net household income can certainly be associated with the relationship between employment status and mental health. In the present model, however, this effect was evident only for female informal caregivers but not for male informal caregivers.

The gender differences may be due to the fact that women showed poorer mental health in the overall sample. Likewise, Lacey, McMunn [21] found effects of informal caregiving on mental health only for women. In accordance with social role distributions, women provide informal care more often than men and use support services less frequently than men [44, 45]. Consequently, women experience more negative consequences from providing informal care. In terms of a dose-response relationship, the number of hours spent providing informal care can be a key factor with regard to the effects of informal caregiving on caregivers’ mental health [12, 29]. Thus, the relationship between employment status and mental health is moderated by income among women due to a combination of heightened vulnerability to economic stressors [46, 47], unique societal expectations [47], and differences in coping mechanisms as well as support systems [44]. Further, women have a higher risk of poor mental health than men, especially during intensive care work [12]. However, in the present study, the degree of complexity of the care tasks was a significant factor in mental health only for male informal caregivers but not for female informal caregivers.

### Strengths and limitations

The current analysis has some strengths and limitations. Only cross-sectional data were analyzed, so no causal relationships could be identified. However, the data were sufficient for considering initial correlations with regard to the moderating effect of net household income on the relationship between employment and care. Although we considered data from 2002 to 2018 in the present analysis of data from the SOEP, we pooled the data from multiple years into a single cross-sectional data set so that we were able to utilize a relatively large sample. In addition, only limited determinants relating to care receivers and the care situation were available in the data on the informal caregivers from the SOEP survey data, and thus, we could not include a large variety of confounding variables in our analyses. This includes also, that there was no differentiated operationalisation of employment status available. However, only the aspect of social causation was considered in the present analysis, but not the opposite effect in the sense of social selection. Nevertheless, the present analysis provided a large, Germany-wide depiction of the situation of informal caregivers. Because we had access to a generally representative population survey (SOEP survey), it was also possible to reach relatives who were responsible for giving care but who have otherwise not been included in surveys of informal caregivers because they have not yet been in contact with the care system.

## Conclusion

In this study, we showed that net household income has a relevant effect on the relationship between informal caregivers’ employment status and their mental health, especially for women. In addition, informal caregivers who also work full-time jobs had better mental health than those who were unemployed or only marginally employed. Therefore, due to the shortage of skilled workers and the importance of the mental health of employed informal caregivers for their productivity at work, it is necessary to create structures that enable employed informal caregivers to achieve work-life-care balance without mental stress.

Future studies should examine the relevance of net household income to informal caregivers’ mental health in more detail, especially focusing on the reciprocal effect between mental health and employment. Thereby, the financial situations of younger informal caregivers who are not yet receiving a retirement pension or similar, as in this sample, should be investigated in particular. It is possible that this group of informal caregivers is exposed to even greater financial pressure and thus greater psychological stress.

## Data Availability

The datasets used and analyzed during the current study are available from the corresponding author on reasonable request.

## References

[1] OECD. Informal carers. 2017.

[2] Federal Statistical Office. 15. koordinierte Bevölkerungsvorausberechnung für Deutschland [15th coordinated population prognosis for Germany]. 2023.

[3] Lisenkova K, McQuaid RW, Wright RE. Demographic change and labour markets. Twenty-First Century Soc. 2010;5(3):243–59.

[4] Bührer C, Hagist C. The effect of digitalization on the labor market. The Palgrave Handbook of managing continuous business transformation. 2017:115 – 37.

[5] Vinarski-Peretz H, Halperin D. Informal caregivers along the work–eldercare axis: a comparative analysis of Australia, England, and Israel. Int J Law Policy Family. 2021;35(1):ebaa015.

[6] Longacre ML, Valdmanis VG, Handorf EA, Fang CY. Work impact and emotional stress among informal caregivers for older adults. Journals Gerontol Ser B: Psychol Sci Social Sci. 2017;72(3):522–31. 10.1093/geronb/gbw027 PMC5926991 27048567

[7] Neubert L, König H-H, Mietzner C, Brettschneider C. Dementia care-giving and employment: a mixed-studies review on a presumed conflict. Ageing Soc. 2021;41(5):1094–125.

[8] Bauer JM, Sousa-Poza A. Impacts of informal caregiving on caregiver employment, health, and family. J Popul Ageing. 2015;8(3):113–45.

[9] WHO. Mental Health Atlas 2011. World Health Organization; 2011.

[10] Bidenko K, Bohnet-Joschko S. Vereinbarkeit Von Beruf Und Pflege: Wie Wirkt Sich Erwerbstätigkeit auf die Gesundheit pflegender Angehöriger aus? [Balancing work and caregiving: Impact of Employment on the health of family caregivers]. Gesundheitswesen. 2021;83(02):122–7. 32645733 10.1055/a-1173-8918

[11] Wang Y-N, Hsu W-C, Yang P-S, Yao G, Chiu Y-C, Chen S-T, et al. Caregiving demands, job demands, and health outcomes for employed family caregivers of older adults with dementia: structural equation modeling. Geriatr Nurs. 2018;39(6):676–82. 29859698 10.1016/j.gerinurse.2018.05.003

[12] Bom J, Bakx P, Schut F, Van Doorslaer E. The impact of informal caregiving for older adults on the health of various types of caregivers: a systematic review. Gerontologist. 2019;59(5):e629–42. 30395200 10.1093/geront/gny137 PMC6850889

[13] Verbakel E, Metzelthin SF, Kempen GI. Caregiving to older adults: determinants of informal caregivers’ subjective well-being and formal and informal support as alleviating conditions. J Gerontol Ser B: Psychol Sci Social Sci. 2018;73(6):1099–111. 10.1093/geronb/gbw047 27130169

[14] Stratmann M, Forsell Y, Möller J, Liang Y. Informal care and the impact on depression and anxiety among Swedish adults: a population-based cohort study. BMC Public Health. 2021;21(1):1–7. 34187429 10.1186/s12889-021-11246-1 PMC8243546

[15] Kumagai N. Distinct impacts of high intensity caregiving on caregivers’ mental health and continuation of caregiving. Health Econ Rev. 2017;7:1–14. 28389976 10.1186/s13561-017-0151-9 PMC5383799

[16] Le DD, Ibuka Y. Understanding the effects of informal caregiving on health and well-being: heterogeneity and mechanisms. Soc Sci Med. 2023;317:115630. 36580861 10.1016/j.socscimed.2022.115630

[17] Pearse R, Connell R. Gender norms and the economy: insights from social research. Fem Econ. 2016;22(1):30–53.

[18] King T, Hewitt B, Crammond B, Sutherland G, Maheen H, Kavanagh A. Reordering gender systems: can COVID-19 lead to improved gender equality and health? Lancet. 2020;396(10244):80–1. 32569582 10.1016/S0140-6736(20)31418-5 PMC7304958

[19] Cascella Carbó GF, García-Orellán R. Burden and gender inequalities around informal care. Investigacion Y Educ en enfermeria. 2020;38(1). 10.17533/udea.iee.v38n1e10 PMC7871478 32124578

[20] Boniol M, McIsaac M, Xu L, Wuliji T, Diallo K, Campbell J. Gender equity in the health workforce: analysis of 104 countries. World Health Organization; 2019.

[21] Lacey RE, McMunn A, Webb E. Informal caregiving patterns and trajectories of psychological distress in the UK Household Longitudinal Study. Psychol Med. 2019;49(10):1652–60. 30205848 10.1017/S0033291718002222 PMC6601356

[22] Federal Ministry for Family Affairs, Senior Citizens, Women and Youth. Who takes care of children, household and the elderly? A dossier on the societal dimension of a private question. 2020.

[23] Federal Statistical Office. Gender Care Gap. Frauen Leisten 43,8% mehr unbezahlte Arbeit als Männer [press release]. 2022 Feb 28.

[24] Lietzmann T, Frodermann C. Gender role attitudes and labour market behaviours: do attitudes contribute to gender differences in employment in Germany? Work. Employ Soc. 2023;37(2):373–93.

[25] Ervin J, Taouk Y, Alfonzo LF, Peasgood T, King T. Longitudinal association between informal unpaid caregiving and mental health amongst working age adults in high-income OECD countries: A systematic review. EClinicalMedicine. 2022;53:101595. 10.1016/j.eclinm.2022.101711 PMC9637877 36353526

[26] Zwar L, König H-H, Hajek A. Gender differences in mental health, quality of life, and caregiver burden among informal caregivers during the second wave of the COVID-19 pandemic in Germany: a representative, population-based study. Gerontology. 2023;69(2):149–62. 35390788 10.1159/000523846 PMC9148891

[27] Brown MJ, Cohen SA. Informal caregiving, poor mental health, and subjective cognitive decline: results from a population-based sample. J Gerontol Nurs. 2020;46(12):31–41. 33232495 10.3928/00989134-20201106-04 PMC8045765

[28] Gérain P, Zech E. Informal caregiver burnout? Development of a theoretical framework to understand the impact of caregiving. Front Psychol. 2019;10:466359. 10.3389/fpsyg.2019.01748 PMC6689954 31428015

[29] Fleitas Alfonzo L, Taouk Y, Emerson E, King T. Impact of informal care on the mental health of caregivers during the COVID-19 pandemic. J Public Health. 2023;45(4):e668–76. 10.1093/pubmed/fdad193 PMC10687605 37786356

[30] Nam I. Financial difficulty effects on depressive symptoms among dementia patient caregivers. Community Ment Health J. 2016;52:1093–7. 27289466 10.1007/s10597-016-0033-3

[31] Chiao CY, Wu HS, Hsiao CY. Caregiver burden for informal caregivers of patients with dementia: a systematic review. Int Nurs Rev. 2015;62(3):340–50. 26058542 10.1111/inr.12194

[32] Goetze H, Brähler E, Gansera L, Schnabel A, Köhler N. Exhaustion and overload of family caregivers of palliative cancer patients. Psychother Psychosom Med Psychol. 2014;65(2):66–72. 25405873 10.1055/s-0034-1385933

[33] Goebel J, Grabka MM, Liebig S, Kroh M, Richter D, Schröder C, et al. The German socio-economic panel (SOEP). Jahrb Natl Stat. 2019;239(2):345–60.

[34] Kroh M, Kühne S, Siegers R, Belcheva V. SOEP-Core-Documentation of sample sizes and panel attrition (1984 until 2016). SOEP Survey Papers; 2018.

[35] Andersen HH, Mühlbacher A, Nübling M, Schupp J, Wagner GG. Computation of standard values for physical and mental health scale scores using the SOEP version of SF-12v2. J Contextual Economics–Schmollers Jahrbuch. 2007;1:171–82.

[36] Liu Y, Dokos M, Fauth EB, Lee YG, Zarit SH. Financial strain, employment, and role captivity and overload over time among dementia family caregivers. Gerontologist. 2019;59(5):e512–20. 31322654 10.1093/geront/gnz099 PMC6857684

[37] Barnett AE. Adult child caregiver health trajectories and the impact of multiple roles over time. Res Aging. 2015;37(3):227–52. 25651570 10.1177/0164027514527834

[38] Chumbler NR, Pienta AM, Dwyer JW. The depressive symptomatology of parent care among the near elderly: the influence of multiple role commitments. Res Aging. 2004;26(3):330–51.

[39] Martire LM, Stephens MAP, Atienza AA. The interplay of work and caregiving: relationships between role satisfaction, role involvement, and caregivers’ well-being. Journals Gerontol Ser B: Psychol Sci Social Sci. 1997;52(5):S279–89. 10.1093/geronb/52b.5.s279 9310100

[40] Hollederer A, Wildner M. Subjektive Gesundheit Und Erwerbslosigkeit in Deutschland auf Basis der EU-SILC-Daten Von 2005 bis 2014 [Subjective Health and Unemployment in Germany: Analysis of 2005–2014 EU-SILC Data]. Gesundheitswesen. 2019;81(12):1082–90. 30463099 10.1055/a-0725-8164

[41] Mossakowski KN. Social causation and social selection. The Wiley Blackwell encyclopedia of health, illness, behavior, and society. 2014:2154-60.

[42] Eberl A, Lang S, Seebaß K. The impact of informal care and employment on the mental health of the caregiver. Sozialer Fortschritt. 2017;66(1):77–96.

[43] Ciccarelli N, Van Soest A. Informal caregiving, employment status and work hours of the 50 + population in Europe. De Economist. 2018;166(3):363–96. 30996393 10.1007/s10645-018-9323-1 PMC6434966

[44] Lüdecke D, Mnich E, Kofahl C. The impact of sociodemographic factors on the utilisation of support services for family caregivers of elderly dependents - results from the German sample of the EUROFAMCARE study. GMS Psycho-Social-Medicine. 2012;9:1–11. 10.3205/psm000084 PMC3488802 23133500

[45] Pinquart M, Sörensen S. Gender differences in caregiver stressors, social resources, and health: an updated meta-analysis. J Gerontol Ser B: Psychol Sci Social Sci. 2006;61(1):33–45. 10.1093/geronb/61.1.p33 16399940

[46] Buffel V, Van de Straat V, Bracke P. Employment status and mental health care use in times of economic contraction: a repeated cross-sectional study in Europe, using a three-level model. Int J Equity Health. 2015;14:1–19. 25889356 10.1186/s12939-015-0153-3 PMC4367872

[47] Li AKC, Nowrouzi-Kia B. Relationships between employment status with self-perceived mental and physical health in Canada. AIMS Public Health. 2024;11(1):236. 38617416 10.3934/publichealth.2024012 PMC11007413

